# Outcomes of arthroscopic tuberoplasty for symptomatic irreparable rotator cuff tendon tear without pseudoparalysis

**DOI:** 10.1016/j.jseint.2022.06.007

**Published:** 2022-07-16

**Authors:** Yaniv Pines, Kevin M. Magone, Erel Ben-Ari, Dan Gordon, Andrew S. Rokito, Mandeep S. Virk, Young W. Kwon

**Affiliations:** Department of Orthopedic Surgery, New York University Langone Orthopedic Hospital, New York, NY, USA

**Keywords:** Shoulder, Rotator cuff tear, Irreparable, Symptomatic, Arthroscopic tuberoplasty, Arthroscopy, Without pseudoparalysis

## Abstract

**Background:**

The purpose of this study is to report the outcomes in patients undergoing arthroscopic tuberoplasty for symptomatic irreparable rotator cuff tear (RCT).

**Methods:**

This is a retrospective cohort study comparing preoperative and postoperative data of patients undergoing arthroscopic tuberoplasty for symptomatic irreparable RCT. Exclusion criteria included open tuberoplasty, concomitant partial RCT repair, glenohumeral arthritis, concomitant ipsilateral extremity fractures, <12 months follow-up, or pseudoparalysis. Demographics, shoulder range of motion (ROM), RCT morphology, re-operation rates, satisfaction and outcome scores were collected from medical records and questionnaires. Outcome scores included Patient Reported Outcome Measurement Information System Upper Extremity (PROMIS), American Shoulder and Elbow Surgeons score (ASES), Subjective Shoulder Value (SSV), and pain Visual Analog Score (VAS).

**Results:**

Out of 28 patients identified between 2012 and 2019, 20 (21 shoulders) were available for follow-up at a mean of 43.3 ± 20.9 months. Mean age was 64.6 ± 8.8 years. Mean PROMIS was 37.7 ± 7.3, ASES was 82.9 ± 13.8, and SVV was 67.1 ± 19.4. VAS with activity decreased from 5.0 ± 2.9 preoperatively to 2.3 ± 2.6 (*P* = .0029). Pre- and post-operative ROM were unchanged. There were 4 failures requiring revision. The remaining 17 patients reported high satisfaction scores (3.4 ± 0.7) and 15 (88.2%) answered “yes” to getting the procedure again, with 3/4 failures stating they would also undergo arthroscopic tuberoplasty again.

**Conclusion:**

Arthroscopic tuberoplasty demonstrates high levels of satisfaction and pain reduction in symptomatic irreparable RCT. In appropriately indicated patients, this treatment should be considered prior to other salvage options.

Treatment of massive or symptomatic irreparable rotator cuff tears can be challenging. Although classically defined as a tear >5 cm or involving more than two tendons, more recent studies suggest that surgical repair of massive rotator cuff tears should consider several additional factors such as fatty infiltration, tendon length of <15 mm measured on magnetic resonance imaging (MRI), retraction beyond the rim of the glenoid, fixed humeral head subluxation, tear at the infraspinatus muscle-tendon junction, and failure of a prior repair.^1^ In fact, rather than undergoing primary repair, some authors advocate a joint-preserving procedure such as débridement, acromioplasty, biceps tenotomy or tenodesis, balloon implantation, graft interposition, superior capsular reconstruction, or tendon transfer in patients with high risk for failure and without glenohumeral arthritis or pseudoparalaysis.^1^^,^^3^^,^^14^

Despite the number of available options, no consensus on optimal management of massive or symptomatic irreparable rotator cuff tears exists, particularly in elderly patients without pseudoparalysis or evidence of glenohumeral arthritis. While some salvage procedures, such as superior capsular reconstruction or tendon transfer, are better suited for younger patients with high functional demand, others including arthroscopic débridement and subacromial decompression may be sufficient for older patients with low functional demands whose primary complaint is pain. These latter options may be effective for pain relief, but do not halt the progression of arthritis and may not be as durable compared to other treatment options.^14^ Other considerations include the associated costs, surgical risk tolerance, complication rates, and the burdens of postoperative rehabilitation.

Alternatively, another previously described surgical option for massive or symptomatic irreparable rotator cuff tears is tuberoplasty. Originally introduced by Fenlin et al, the principle of the tuberoplasty procedure is to relieve subacromial impingement by reshaping the greater tuberosity to create a smooth articulation between the greater tuberosity and the undersurface of the acromion during shoulder abduction.^4^ Importantly, the coracoacromial (CA) arch, which acts as a passive stabilizer to anterior and superior humeral head displacement, is preserved during this procedure. Tuberoplasty was originally described as an open procedure, but several subsequent studies have since reported outcomes of an arthroscopic technique. However, the available data in the literature in terms of patient satisfaction, return to work, and failure rates are still sparse.

Therefore, the aim of this study is to present our experience with arthroscopic tuberoplasty in patients with massive or symptomatic irreparable rotator cuff tears without pseudoparalysis. We hypothesize that this procedure will show improvement in clinical outcomes and pain with activity.

## Materials and methods

### Study design

This is a retrospective case series of patients undergoing arthroscopic tuberoplasty for massive or symptomatic irreparable rotator cuff tears without pseudoparalysis with a minimum of 12 months follow-up period. This study was approved by our institutional review board and is in compliance with Health Insurance Portability and Accountability Act protocols.

### Subject eligibility and enrollment

Eligible subjects were identified by chart review of cases performed by the senior author at our institution between September 2012 and November 2019. A total of 28 patients who underwent arthroscopic tuberoplasty were identified. All patients were evaluated preoperatively with an MRI that demonstrated a massive or irreparable rotator cuff tear with retraction to at least the mid humeral head and no signs of advanced osteoarthritis. Preoperatively, all patients demonstrated the ability to actively elevate their arm to at least 120 degrees. Six patients were unable to be contacted for follow-up and two declined to participate leaving a total of 21 shoulders (20 patients) in the final analysis. We excluded subjects who had tuberoplasty by open approach, concomitant partial rotator cuff repair, glenohumeral arthritis, concomitant ipsilateral extremity fractures, follow-up of less than 12 months, or those unwilling to participate.

### Operative technique

**Figure 1:**
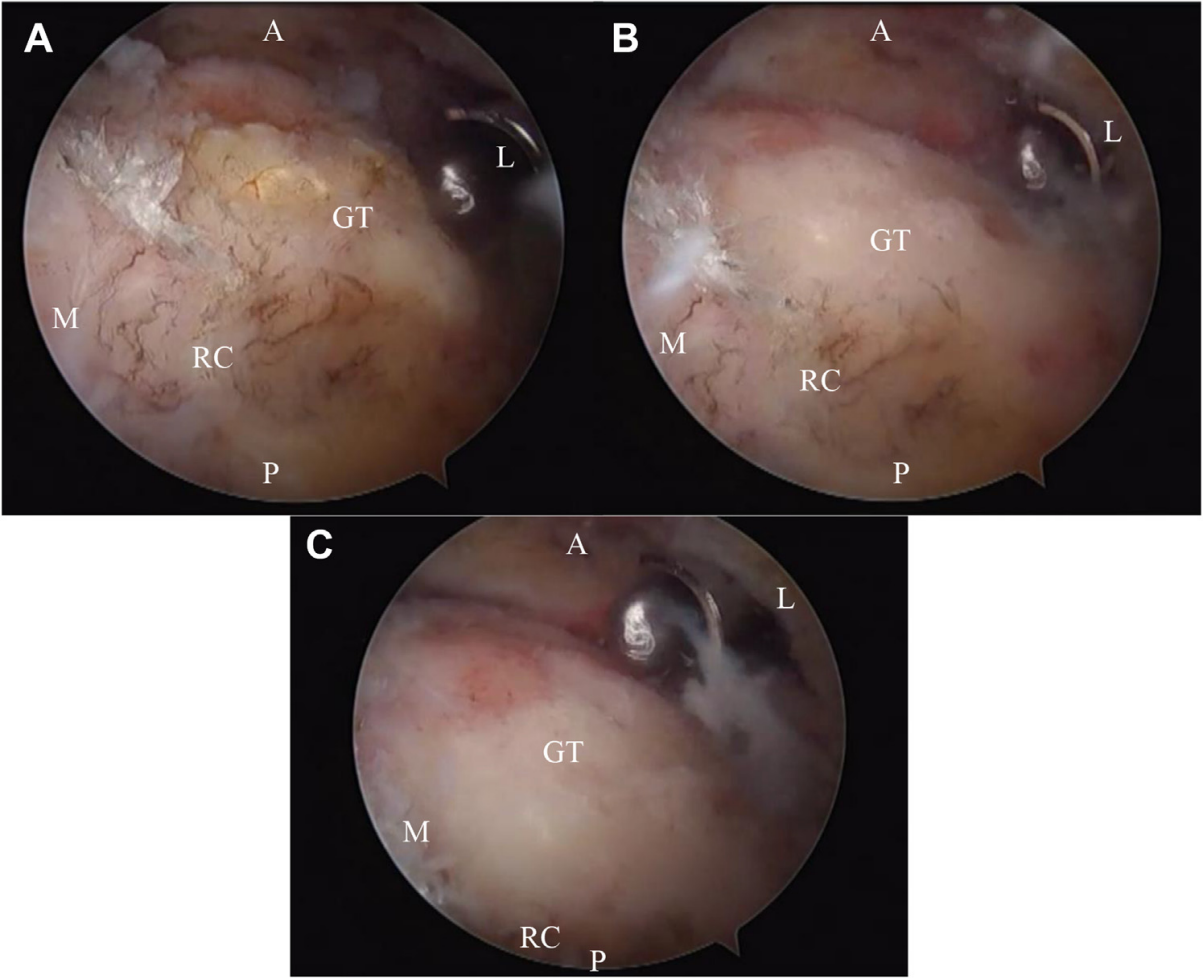
Right shoulder, beach chair position, viewing from posterolateral portal with 30° arthroscope.

All procedures were performed in the beach chair position under general or brachial plexus anesthesia. Standard posterior, lateral, and anterior arthroscopy portals were used. During arthroscopy, the glenohumeral joint was evaluated and if needed, a partial synovectomy of the glenohumeral joint was performed to débride any inflamed tissue. The rotator cuff tendon tears were evaluated and, in all cases, the tendon was mobilized for possible repair. When the tendon was deemed irreparable despite aggressive mobilization, tuberoplasty was performed (Fig. 1). In order to protect the CA arch, the CA ligament and the acromion were maintained. Tuberoplasty was performed with an arthroscopic burr such that the greater tuberosity was co-spherical with the humeral head. This process started from the posterior edge of the bicipital groove and was extended posteriorly until the insertion of the remaining rotator cuff tendon on the posterior portion of the greater tuberosity was encountered. After tuberoplasty was complete, the shoulder was abducted to ensure that the tuberosity no longer impinged against the lateral border of the acromion. For patients who had previously undergone arthroscopic repair, the authors removed the suture material and suture anchors as needed to obtain a smooth spherical surface on the tuberosity. Concomitant arthroscopic biceps tenodesis was performed in 14 of the 21 shoulders where a tear of the long head of the biceps (LHB) tendon, instability, tenosynovitis, or a positive clinical exam for LHB were noted. Concomitant AC joint resection was performed in 16 of 21 shoulders with preoperative joint tenderness and intraoperative arthritic degeneration.

### Rehabilitation

Immediately after the procedure, all patients were provided with a sling which was to be worn at their discretion only. At one week after the surgery, all patients proceeded with supervised therapy for active and passive motion. At 5-6 weeks, the rehabilitation protocol was advanced to include resistive strength training.

### Clinical evaluation

Preoperatively, shoulder active range of motion (ROM) was documented, including forward flexion (FF), external rotation at the side (ERs), and internal rotation to the posterior (IRp). Shoulder internal rotation was measured by vertebral segments and converted to the following discrete assignments for statistical evaluation: 0° = 0, hip = 1, buttock = 2, sacrum = 3, L5-L4 = 4, L3-L1 = 5, T12-T8 = 6, T7 or higher = 7.^21^ Subjective pain with activity was recorded using a Visual Analogue Scale (VAS), with 0 being no pain in the affected shoulder and 10 being the worst possible pain.

Postoperatively, patients completed questionnaires either in person or over telephone. Functional outcomes included the Patient Reported Outcome Measurement Information System Upper Extremity Computer Adaptive Test (PROMIS UE CAT), American Shoulder and Elbow Surgeons (ASES) score, Subjective Shoulder Value (SSV), and pain VAS. PROMIS Pain Interference and Pain Intensity scores, in addition to PROMIS Global 10 Physical Health and Mental Health, were also completed. PROMIS instruments are scored are on the T-score metric, with the mean of 50 and standard deviation of 10 set to equal the mean of the US general population, and scores ranging from approximately 15-60.^6^ A higher score indicates more of that domain being measured; a higher UE CAT indicates higher upper extremity physical function and, for instance, a higher score in Global 10 Physical Health indicates higher levels of physical health.^6^

Follow-up ROM data were collected using subjective patient assessment. If unable to be examined in person, subjects were sent pictorial diagrams and asked to choose the highest level they could reach without assistance in FF, ERs, and IRp. Previous literature has demonstrated suitable reliability of subjective patient ROM assessment using these diagrams.^2^^,^^17^^,^^25^

In addition, subjects were asked about level of satisfaction, return to work, and return to sports. Satisfaction was evaluated both by using a scale from 0-4 (0 = extremely dissatisfied, 1 = dissatisfied, 2 = neither dissatisfied nor satisfied, 3 = satisfied, and 4 = extremely satisfied) and a yes/no response to undergoing the surgery again. Clinical failures were defined as any revision surgery in the ipsilateral shoulder after undergoing arthroscopic tuberoplasty.

### Preoperative radiographic evaluation

Preoperatively, 11/21 (47.6%) shoulders had available radiographs. The acromiohumeral interval (AHI) was measured in millimeters (mm) and used to calculate the preoperative Hamada classification.

### Statistical analysis

Data were evaluated for normality using the Shapiro–Wilk test and those with normal distributions were analyzed using the independent and paired t test. Nonparametric analysis was performed with the Wilcoxon signed rank test to compare data that was not normally distributed. Nonparametric Spearman's rank correlation was used to determine the relation between preoperative variables (age at time of surgery, operative side, gender, preoperative AHI, and active mobility) and postoperative PROMIS UE, ASES, and VAS pain scores. Statistical analyses were performed using RStudio version 1.2.1335 (RStudio, PBC, Boston, MA, USA), with a *P*-value below .05 considered statistically significant.

## Results

### Demographics

A total of 21 shoulders from 20 patients were included for the final analysis. The mean age at time of surgery was 64.6 ± 8.8 years with 15 (71.4%) males and 6 (28.6%) females. The mean follow-up period was 43.3 ± 20.9 months. All but seven (66.7%) procedures were on the right shoulder and the dominant side was operated on for 13/21 shoulders (61.9%). One patient underwent bilateral arthroscopic tuberoplasty. Four subjects had a previous history of rotator cuff repair, ranging between 1 and 2 procedures (mean of 1.3 procedures). Average AHI was 7.5 ± 5.7 mm; average Hamada grade was 1.7 (range of 1-3). On preoperative MRI, 4 (19.0%) shoulders demonstrated a tear of only the supraspinatus tendon, 14 (66.7%) had a tear of both supraspinatus and infraspinatus tendons, one (4.7%) had a tear of both supraspinatus and subscapularis, and 2 (9.5%) had tears of all three tendons. Complete demographics are shown in Table I.

**Table I:** Demographics.

Number of subjects (n)	20 (21 shoulders)
Age at surgery (years)	64.6 ± 8.8
Follow-up (months)	43.3 ± 20.9
Smoking status	
Never smoker	14/21 (66.7%)
Former smoker	6/21 (28.6%)
Current smoker	1/21 (4.8%)
Gender	
Male	15/21 (71.4%)
Female	6/21 (28.6%)
Operated side	
Right	14/21 (66.7%)
Left	7/21 (33.3%)
Dominant side	13/21 (61.9%)
Previous surgery	4/21 (19.0%)
Number of previous surgeries	
Overall	0.2 (0-2)
Subjects with previous surgery	1.3 (1-2)
Cuff involvement	
SS	4 (19.0%)
SS + IS	14 (66.7%)
SS + SSc	1 (4.7%)
SS + IS + SSc	2 (9.5%)

*SS*, Supraspinatus; *IS*, Infraspinatus; *SSc*, Subscapularis.

### Range of motion

Preoperative and postoperative active range of motion is shown in Table II. Preoperatively, the mean FF was 155.2° ± 33.9, ERs was 46.4° ± 12.8, and IRp was L4-L5 ± 1.5. Postoperatively, FF, ERs, and IRp were 153.8° ± 38.3, 57.6° ± 29.3, and L1-L3 ± 1.2, respectively. There were no significant changes in any planes of motion.

**Table II:** Active shoulder range of motion before and after arthroscopic tuberoplasty.

ROM	Preoperative Mean (± standard deviation)	Postoperative Mean (± standard deviation)	*P* value
FF (°)	155.2 ± 33.9	153.8 ± 38.3	.94
ERs (°)	46.4 ± 12.8	57.6 ± 29.3	.13
IRp	L4-L5 ± 1.5	L1-L3 ± 1.2	.09

*ROM*, range of motion; *FF*, forward flexion; *ERs*, external rotation at side; *IRp*, internal rotation to posterior.

### Clinical outcomes

Postoperatively, the mean PROMIS UE score was 37.7 ± 7.3. PROMIS Global 10 Mental and Physical Health was 52.8 ± 9.4 and 49.6 ± 8.7, respectively. The mean ASES score was 82.9 ± 13.8. To measure pain in the operative shoulder, VAS, PROMIS Pain Interference and PROMIS Pain Intensity were completed. In comparison to preoperative condition, VAS was significantly decreased at the latest follow-up from 5.0 ± 2.9 to 2.3 ± 2.6 (*P* = .0029). Mean PROMIS Pain Interference and Pain Intensity was 51.7 ± 9.4 and 42.1 ± 10.3, respectively. Patients' final mean SSV was 67.1 ± 19.4. Complete postoperative clinical outcomes are shown in Table III.

**Table III:** Postoperative clinical outcomes.

Outcome score	Mean (± standard deviation)
PROMIS Upper Extremity	37.7 ± 7.3
PROMIS Pain Interference	51.7 ± 9.4
PROMIS Pain Intensity	42.1 ± 10.3
PROMIS Global 10- Mental Health	52.8 ± 9.4
PROMIS Global 10- Physical Health	49.6 ± 8.7
ASES	82.9 ± 13.8
VAS pain with activity	2.3 ± 2.6 (vs 5.0 ± 2.9 preoperatively, *P* = .0029)
SSV	67.1 ± 19.4

*PROMIS*, Patient Reported Outcome Measurement Information System; *ASES*, American Shoulder and Elbow Surgeons score; *VAS*, visual analogue score; *SSV*, subjective shoulder score.

### Return to sport

Out of 20 subjects, 6 were playing recreational sports prior to undergoing arthroscopic tuberoplasty. Four of these 6 (66.7%) were able to return to sport but only one was able to return at the same or higher level. One patient cited pain and weakness in the operative shoulder as the reason for not returning to sport, while the other cited lifestyle changes.

### Return to work

Of the 20 subjects, 12 were working prior to the procedure, 5 of which were manual laborers. Ten of these 12 (83.3%) were able to return to work after arthroscopic tuberoplasty. Of note, 3/5 (60.0%) of the manual laborers were also able to return to work. Both patients who did not return to work were manual laborers; one decided to retire and the other cited disability related to his operative shoulder as the reason for not working.

### Failure rate

Overall, there were 4 (19.0%) clinical failures, defined as the return of pain and weakness of the operative shoulder requiring additional surgery. One of these failures initially reported good clinical outcome, but then fell at 14 months after the procedure and was no longer able to elevate the arm. All patients with failures were treated with a reverse total shoulder arthroplasty (rTSA). For these patients, symptoms began to recur at a mean of 8.8 ± 11.7 months post-tuberoplasty and revision surgery occurred 14.1 ± 13.6 months post-tuberoplasty.

### Satisfaction

In the 17 shoulders without clinical failure, 15 (88.2%) reported being satisfied or extremely satisfied with the procedure, 1 (5.9%) reported being neither satisfied nor dissatisfied, and 1 (5.9%) was extremely unsatisfied. Fifteen patients (88.2%) reported that they would undergo the procedure again if necessary.

As expected, the lowest satisfaction scores were reported from the group with clinical failures. Interestingly, 3 of these 4 patients who required revision surgery stated that they would still proceed with the arthroscopic tuberoplasty procedure again in a similar situation, and did not regret their decision despite the failure.

### Correlations

Using nonparametric Spearman's rank correlation, there was no statistically significant correlation between any preoperative variable (age at time of surgery, laterality, gender, preoperative AHI, and active mobility) and postoperative outcomes (PROMIS UE, ASES, and VAS pain scores). In addition, patient outcome parameters, including failure rates, in patients with and without concomitant biceps tenodesis also did not demonstrate any statistically significant differences.

## Discussion

This study demonstrates promising clinical outcomes after arthroscopic tuberoplasty for appropriately chosen and counseled patients with massive or symptomatic irreparable rotator cuff tear. Despite no change in shoulder active ROM, pain levels were significantly improved at the latest follow-up. Among those who did not undergo revision surgery, satisfaction was high and most patients in this group agreed to undergo the procedure again if recommended. However, there were 4 clinical failures all requiring rTSA.

The treatment of massive or symptomatic irreparable rotator cuff tears continues to present a major dilemma for orthopedic surgeons. Numerous salvage procedures have been reported but there is still no single gold-standard procedure to treat this pathology while preserving the native shoulder joint. For elderly patients, subacromial decompression with acromioplasty is an option for pain relief but its efficacy may deteriorate over time as a result of violation of the coracoacromial ligament.^19^^,^^20^^,^^23^ Previous studies have shown that the coracoacromial ligament prevents superior migration of the humeral head and maintains the force coupling system in the shoulder which, in turn, can prevent the progression into rotator cuff arthropathy).^5^^,^^8^^,^^11^^,^^13^^,^^26^ As an alternative, Fenlin first developed the open tuberoplasty in 2002, which serves as a decompressing procedure without violating the CA arch.^4^ At a mean follow-up period of 27 months in 20 shoulders, the authors reported significant improvement in pain and function, but residual weakness in external rotation. Schiebel et al^16^ subsequently developed an arthroscopic technique termed a reverse arthroscopic subacromial decompression and reported pain and functional improvement in 23 patients at a mean follow-up of 40 months. Several small studies have since also reported similarly promising results.^10^^,^^15^^,^^20^ Most recently, Park et al^15^ conducted the longest study to date, with a mean follow-up period of 8 years after arthroscopic tuberoplasty. Despite evidence of superior migration of the humeral head, clinical outcomes were maintained.

Matsen et al describe a similar “smooth and move” procedure, reporting improvements in Simple Shoulder Test scores, abduction and FF at 6 weeks postoperatively.^7^^,^^12^ These studies report overall low rates of complications, clinical failures or revision surgery (between 0.0% and 5.4%). Another promising option is subacromial balloon spacer implantation. Like a tuberoplasty, this is a low morbidity procedure that can be done arthroscopically with concomitant débridement, bursectomy or biceps tenotomy/ tenodesis. Several studies published recently have shown favorable results.^18^^,^^22^^,^^24^ Notably, Stewart et al^18^ conducted a systematic review that included 12 studies for final analysis showing improvements in mean Constant Scores from 18.5 to 49.6 at an average of 22.9 months after implantation. These studies also describe similarly low rates of complications, revision surgeries, or clinical failures.

In our cohort, active ROM in all planes did not change significantly after arthroscopic tuberoplasty compared to preoperative values. However, this was not the case in previous studies. Verhelst et al, for example, found that FF and abduction were increased at 38 months post-tuberoplasty. Lee et al also reported significant improvement in FF and ER at 90 degrees of abduction at 40 months postoperatively. Park et al found results more consistent with ours, with only FF showing significant improvement at 98 months. These disparities may be due to ROM being limited by pain and not necessarily the functional ability of the rotator cuff.

Despite a lack of improvement in active range of motion, patients experienced significant relief of pain. In addition, PROMIS Pain Intensity and Pain Interference scores were relatively low. The mean PROMIS Upper Extremity score (which measures only function but not pain) was relatively low, indicating that patients continue to have sub-optimal function. These findings mirror the lack of ROM improvement. However, mean ASES scores were measured to be high. The discrepancy between these two outcome measures may be two-fold; PROMIS UE and ASES measure different combinations of patient health domains and place different weights on each domain in calculating total scores. PROMIS UE measures only upper extremity function, while ASES measures both pain and function and assigns both a weight of 50% for the total score. When calculating a total ASES score, a lower pain subscore will translate into a higher total score, which is the case in our series. These findings are consistent with the previous literature.^4^^,^^10^^,^^15^^,^^16^^,^^20^

There were no statistically significant correlations between any preoperative variable (age at time of surgery, laterality, gender, preoperative AHI, and active mobility) and postoperative outcomes (PROMIS UE, ASES, and VAS pain scores). Overall, in patients without clinical failure, most reported being satisfied or extremely satisfied and that they would undergo the procedure again if recommended. In addition, most of those who were working or playing sports preoperatively were able to return. There were 4 patients (19.0%) who underwent subsequent rTSA.

For massive or symptomatic irreparable rotator cuff tears, a number of salvage options have been proposed, including débridement, acromioplasty, biceps tenotomy or tenodesis, balloon implantation, graft interposition, superior capsular reconstruction, tendon transfer as well as rTSA. As each of these procedures has its respective advantages and disadvantages, no consensus exists regarding optimal management. Therefore, despite our relatively high reoperation rates, arthroscopic tuberoplasty is a reasonable option in lower demand patients with massive or symptomatic irreparable rotator cuff tears without pseudoparalysis. While providing relatively high rates of satisfaction, the procedure is minimally invasive with low morbidity and complication rate and a less intensive rehabilitation.^9^ Moreover, it is important to note that SSV scores between those with and without clinical failure were not statistically significant at latest follow-up. Therefore, a prior tuberoplasty may not adversely affect the outcome of a subsequent prosthesis or salvage surgery. Due to the variety of treatment options available for massive or symptomatic irreparable rotator cuff tears, the importance of a patient-centered and shared decision-making approach cannot be overemphasized. Optimal candidates for arthroscopic tuberoplasty may be elderly patients without pseudoparalysis or glenohumeral arthritis who have lower functional demand of the arm. In these patients, the goal of this procedure would be to alleviate pain and prevent a salvage type procedure.

## Limitations

This study has several limitations. Like all retrospective reviews, the data may suffer from recall and selection bias. In addition, the cohort is a small number of patients undergoing a relatively uncommon procedure by a single surgeon at our institution. Therefore, the clinical results may not be widely applicable to other patient populations. However, our study design and patient population are similar to those of previous studies reporting outcomes of tuberoplasty. Most importantly, preoperative outcome scores were lacking which prevented demonstration functional improvement associated with the procedure. Future studies should include a longer follow-up period with a control group to mitigate placebo effects in addition to postoperative imaging to assess for superior humeral migration.

## Conclusion

Arthroscopic tuberoplasty has shown promising results in patients with massive or symptomatic irreparable rotator cuff tears without pseudoparalysis or glenohumeral arthritis, leading to high levels of satisfaction, significant pain reduction, and encouraging rates of return to work. Therefore, for appropriate patients, this treatment should be considered prior to other salvage or prosthesis procedures.

## Disclaimers

Funding: No funding was disclosed by the authors.

Conflicts of interest: Young W Kwon: Has consulting relationship with DJO Surgical. The other authors, their immediate families, and any research foundation with which they are affiliated have not received any financial payments or other benefits from any commercial entity related to the subject of this article.
